# Future Directions for Developing Non-dopaminergic Strategies for the Treatment of Parkinson’s Disease

**DOI:** 10.2174/1570159X21666230731110709

**Published:** 2023-08-04

**Authors:** Daniel J. van Wamelen, Valentina Leta, K. Ray Chaudhuri, Peter Jenner

**Affiliations:** 1 Department of Neuroimaging, Institute of Psychiatry, Psychology & Neuroscience, King’s College London, London, United Kingdom;; 2 Department of Basic & Clinical Neuroscience, Institute of Psychiatry, Psychology & Neuroscience, King’s College London, London, United Kingdom;; 3 Parkinson Foundation Centre of Excellence at King’s College Hospital NHS Foundation Trust, London, United Kingdom;; 4 Department of Neurology, Centre of Expertise for Parkinson & Movement Disorders, Donders Institute for Brain, Cognition and Behaviour, Radboud University Medical Center, Nijmegen, the Netherlands;; 5 School of Cancer & Pharmaceutical Sciences, Institute of Pharmaceutical Science, King’s College London, London, United Kingdom

**Keywords:** Parkinson’s disease, pharmacology, non-dopaminergic, drug targets, serotonin, acetylcholine, GABA

## Abstract

The symptomatic treatment of Parkinson’s disease (PD) has been dominated by the use of dopaminergic medication, but significant unmet need remains, much of which is related to non-motor symptoms and the involvement of non-dopaminergic transmitter systems. As such, little has changed in the past decades that has led to milestone advances in therapy and significantly improved treatment paradigms and patient outcomes, particularly in relation to symptoms unresponsive to levodopa. This review has looked at how pharmacological approaches to treatment are likely to develop in the near and distant future and will focus on two areas: 1) novel non-dopaminergic pharmacological strategies to control motor symptoms; and 2) novel non-dopaminergic approaches for the treatment of non-motor symptoms. The overall objective of this review is to use a ‘crystal ball’ approach to the future of drug discovery in PD and move away from the more traditional dopamine-based treatments. Here, we discuss promising non-dopaminergic and ‘dirty drugs’ that have the potential to become new key players in the field of Parkinson’s disease treatment.

## 1 INTRODUCTION

In 1817, James Parkinson in his essay on the Shaking Palsy provocatively said “… Until we are better informed respecting the nature of the disease, the employment of internal medicines is scarcely warrantable” [1]. He might have been less scathing had he foreseen the discovery of the loss of nigral dopaminergic neurons and the depletion of striatal dopamine content that characterises the disease that now bears his name. The subsequent introduction of levodopa revolutionised the treatment of Parkinson’s disease (PD) and the lives of individuals suffering from the illness [2-4]. This in turn led to the dopaminergic revolution with the development of dopamine agonists and adjunct treatments aimed at extending the actions of levodopa.

However, James Parkinson would have felt vindicated by the state of play in advancing the treatment of PD some 200 years later as significant unmet needs remain in relation to motor and non-motor symptomatology, and to the palliative stages of the illness [5]. We have developed a reliance on dopaminergic approaches to treatment at the expense of almost every other potential pharmacological route with perhaps the exceptions of the anticholinergic drugs (although now largely contraindicated for use in Parkinson’s) and the use of amantadine and amantadine derivatives. Yet, we are faced with massive pharmacological challenges that need to be overcome to deal with the levodopa unresponsive motor and non-motor symptoms of PD. The reality is that while we can effectively control bradykinesia and rigidity in the early stages and improve voluntary movement, we still struggle to control tremor, postural, and gait problems as well as a plethora of non-motor symptoms (Table **1**). This has led to suboptimal overall care and is evidenced by surveys where the top 10 troublesome and bothersome symptoms are dominated by non-dopamine-responsive symptoms [6].

We have yet to fully overcome the loss of efficacy of levodopa with disease progression or the onset of motor complications, including troublesome dyskinesia [7-9]. Moreover, the general adverse event profile of dopaminergic drugs in the form of impulse control disorders, excessive daytime sleepiness, and neuropsychiatric disturbances, creates further problems. There has been little enthusiasm for moving outside the dopaminergic arena to meet the challenges associated with treating motor and non-motor components of PD, even though current evidence suggests that much of what fails to be alleviated by classic medication is non-dopaminergic in origin.

Parkinson would probably have had a field day when it came to the treatment of what for a long time was the elephant in the room, the non-motor symptoms (NMS) of PD, some of which have been described by him and directed therapy towards leeches, blisters, arsenic, and curare [10]. Recent times have seen an explosion in the description of NMS, including NMS dominant subtypes of PD and the development of validated clinical rating scales, and the general phenomenology of these very common components of the illness. The realisation that NMS can precede the onset of motor signs and the effect that they have on the quality of life of patients with Parkinson’s disease [11, 12] has brought them to the fore as an unmet need in pharmacological treatment. Most notable is the inadequacy of dopaminergic therapy in controlling many NMS that strongly suggests that their pathophysiology is non-dopaminergic in origin and highlights the need for other approaches to their treatment [13]. Surprisingly, although the interest in the treatment of NMS is increasing [14], many of the pathways involved remain unexplored, and new pharmacological objectives need to be defined.

## 2 MOVING THERAPY TO A NEW ERA

Before moving to a discussion on the direction that novel pharmacological approaches should take, it is necessary to clarify why this is necessary. There are two main arguments that need to be accepted.

First, it is clear that dopaminergic medications do not control all motor symptoms of PD. A good example is our recent review of the underlying basis of treatment-resistant ‘off’ periods [15]. While ‘off’ time and ‘wearing off’ can be reduced by alterations in the extent of oral dopaminergic medication, they cannot be abolished and remain to a considerable degree. Similarly, non-oral therapies, including rotigotine transdermal administration, apomorphine subcutaneous infusion, and intraduodenal levodopa infusion, to maximise the continuity of dopaminergic stimulation, reduce ‘off’ time but fail to provide continuous ‘on’ periods. A detailed analysis of the reasons for this has led to the conclusion that resistant ‘off ‘periods have a non-dopaminergic basis [15]. More specifically, ‘wearing off’ may be dopaminergic in nature and respond to continuous delivery therapies, but additional unpredictable ‘off’ periods may exist that are non-dopaminergic in origin and, as such, would not respond to dopaminergic therapies. The same extends to dyskinesia where changes in basal ganglia circuitry beyond the ‘standard’ dopaminergic inputs are involved in PD [16, 17], including changes to glutamatergic, noradrenergic, and serotonergic pathways [17, 18]. Simply applying a ‘one-size-fits-all’ dopaminergic approach would therefore not be able to address all ‘off’ periods and multimodal approaches, thus tackling multiple neurotransmitter systems would be required.

Second, it is equally obvious that dopaminergic medication does not control all non-motor symptoms. If it did then the prevalence of such symptoms in the treated patient population would be far lower than currently reported. Recent reviews have demonstrated the extent to which a wide range of non-motor symptoms respond to dopaminergic therapies [14, 19, 20]. A broad-brush interpretation would be that some non-motor symptoms respond, particularly those that relate to cognitive, autonomic, and sensory aspects of non-motor fluctuations, but none are fully controlled. Similarly, in recent publications examining the effect of device-aided therapies on non-motor symptoms, including apomorphine subcutaneous infusion, intraduodenal levodopa infusion, and deep brain stimulation, again there is some diminution of some non-motor symptoms but not all and none are abolished [21-23]. This situation is reflected by a wide array of pharmacological treatments that have been used, or have been proposed, for NMS in PD. A specific example includes the broad strategies that have been used to address neuropsychiatric symptoms in PwP; evidence exists regarding the use of dopamine agonists, selective serotonin, and serotonin-norepinephrine reuptake inhibitors for the treatment of depressive symptoms, and clozapine, quetiapine, and pimavanserin may be useful for psychosis [24]. Analogous to the situation mentioned above in relation to non-dopaminergic aspects of motor fluctuations, also for NMS, mainly a dopaminergic approach is unlikely to improve the quality of life in PwP in the long term.

If it is accepted that both dopaminergic and non-dopaminergic mechanisms contribute to both motor and non-motor symptoms of PD, what would be the way forward? First, there is a need to accept the widespread nature of the pathological and biochemical changes that underlie PD [25, 26], and which involve multiple neuronal systems that are not dopaminergic in nature. In the brain, it is difficult to find a neuronal system untouched by the pathology of PD, and almost every neurotransmitter that can be named is altered either directly through cell death or indirectly as an adaptive change to neuronal loss [27-30]. Within the basal ganglia, the primary pathology is certainly the loss of nigral dopaminergic input to the striatum but that has knock-on effects on the circuitry controlling voluntary movement, and alterations occur in a whole range of neurotransmitters associated with the striato-thalamo-cortical loops [27-30]. This is in addition to the loss of inputs from primary pathological change elsewhere that, for example, alters noradrenergic and serotoninergic control of the basal ganglia [29, 31, 32]. This indicates a level of complexity when attempting to locate targets for non-dopaminergic drug treatments, but it also offers the opportunity to move beyond the dopaminergic system.

As such, PD is now considered a multi-symptomatic multi-neurotransmitter disorder associated with a range of motor and non-motor symptoms [25, 33-36]. Many of these symptoms are largely or partly unresponsive to dopamine-based therapies [14, 20, 37, 38], and alternative, adjunctive, treatment should be sought.

## 3 NEUROTRANSMITTER-BASED STRATEGIES

Accepting that the changes that occur in PD, both from a pathological and biochemical point of view, are far more widespread than dopaminergic neurotransmission and affect multiple neuronal pathways and neurotransmitters, the obvious pharmacological way forward is to manipulate those systems to assess the potential efficacy in controlling both motor and non-motor symptoms. As a result, there has been a systematic pharmacological approach to altering the activity of a wide range of neurotransmitters in both normal animals and in several experimental models of PD by systemic and focal administration. This has been coupled with studies aimed at lesioning specific neuronal targets in the brain that decrease the levels of neurotransmitters known to be affected in PD both in isolation and in combination with lesions of nigral dopaminergic neurons. This has not proved easy or amazingly successful as discussed below.

An immediate pharmacological question is whether more can be gained from exploiting the dopaminergic system. Levodopa has already been in use for a long time and a range of adjuncts aimed at potentiating the effects of the drug through enzyme inhibition together with several dopamine agonist drugs, largely aimed at D2 receptors, and technologies that allow intraduodenal, subcutaneous, and transdermal administration, exist. In recent times, new introductions have provided small but important steps forward in treatment, and it could be argued that there is little benefit to be gained over and above what is already available. However, from a pharmacological perspective, there has been little exploitation of the presence of five dopamine receptor subtypes in almost all the nuclei of the basal ganglia [39]. Notably, there has been no successful development of a selective agonist or positive allosteric modulators that bind to D1 receptors outside of the natural ligand site and enhance the signal of dopamine on the receptor [40], and that are devoid of intrinsic activity for D1 dopamine receptors, which control the activity of the direct striatal output pathway, exhibit antiparkinsonian activity, and are involved in dyskinesia expression [41-43]. There are also further on-going attempts to develop D1 agonist drugs, but previous attempts have failed due to concerns over efficacy and tolerance issues [41, 44]. D3 receptor agonists have been avoided over concerns that these receptors are implicated in impulse control disorders and somnolence [45, 46], but again, there has been little clinical development in relation to PD with more interest in areas related to psychiatry. Exploitation of D4 and D5 receptors has been limited, and there has only been passing interest in general in partial agonists, inverse agonists, and antagonists [47-49]. It may be that our enthusiasm has been tempered by a failure to surpass the undeniable efficacy of levodopa. Future direction is more likely to focus on the area of gene therapy with viral vectors being used to introduce a number of genes relevant to dopamine and levodopa metabolism.

In general, attempts to manipulate neurotransmitter systems relevant to the pathology and biochemistry of PD have yielded little that has translated into clinically effective treatments. The transmitters studied include noradrenaline, serotonin, histamine, acetylcholine, glutamate, GABA, and adenosine. While effects on motor and NMS have been shown in preclinical models of the illness, subsequent clinical studies have either failed to show efficacy or have uncovered adverse events not seen in the preclinical studies; this will be discussed in more detail at the end of this review. However, it is worth looking at some examples in more detail to understand the underlying challenges that have impaired progress. As a consequence, a discussion of the possibilities of manipulating cholinergic, GABAergic, and glutamatergic transmission is given below.

## 4 AN ILLUSTRATION OF THE PROBLEMS

In the later stages of PD, alterations in cholinergic function occur in cortical regions, the amygdala, and the hippocampus, leading to cognitive decline and dementia, and like other dementias, these can be treated to some degree with cholinesterase inhibitors [28]. Similarly, changes in cholinergic function in the pedunculopontine nucleus may contribute to gait and freezing episodes and may respond to inhibitors of cholinesterase [50]. But it is the dysfunction of the cholinergic system in the basal ganglia that has received scant attention in recent times. The cholinergic system has an important and sometimes underrated contribution to the motor symptoms of PD, and it has been identified as a specific subtype by some [51] within the spectrum of PD. Cholinergic receptors can be divided into two classes; in the striatum, nicotinic receptors are located pre-synaptically on the terminals of dopaminergic neurons, while muscarinic receptors have a wider distribution and are present on almost all striatal cell bodies and the terminals of input neurons [31, 52, 53]. Overall attempts to use nicotine and nicotinic agonists to alter dopamine release have been unsuccessful, perhaps not surprisingly as the number of presynaptic terminals declines with disease progression [54, 55]. Muscarinic antagonists (generally called anticholinergics) provide useful actions in controlling some motor components of PD, but their use is avoided because of their adverse effects leading to worsening cognition as well as gait and other non-motor symptoms, notably in older patients [55]. These largely reflect the non-selective actions of this older drug class at muscarinic receptors, of which there are five distinct subtypes (M1-M5), all inhibited by current anticholinergics. Surprisingly, there has been little interest in developing subtype-selective muscarinic receptor antagonists for PD even though these might exhibit useful clinical efficacy without the side-effect profile of current drugs. Given that there are different anatomical locations for muscarinic receptor subtypes in the striatum and differences in the distribution of subtypes between brain areas (for example striatum *versus* cortex) [52], this would seem an obvious road to go down, but it is probably blighted by the track record of older drugs and concerns that efficacy may not match that of dopaminergic approaches to treatment. The most surprising aspect of this is that sub-type selective muscarinic antagonists, for example, targeting the M1 receptor subtype, are already available for treating various peripheral disorders, but these have not been similarly applied to the central nervous system; also, other available medications have not been developed further due to side effects, such as xanomeline [56]. Whether repurposed subtype-selective muscarinic receptor antagonists would be useful in treating any NMS of PD seems to have been almost totally ignored [25].

The other major omission from potential non-dopaminergic drug manipulation of basal ganglia function relates to the role of GABAergic neurons and GABAergic receptors. Given that the major striatal output pathways are GABAergic in nature and that the balance of activity between the direct and indirect output pathways is accepted as being key to the control of voluntary movement and is also implicated in the genesis of dyskinesia, it seems strange that this is a pharmacological target that has been largely ignored [9, 30, 57, 58]. There have been few recent attempts to modulate GABAergic transmission or to alter the function of GABA-A or B receptors or benzodiazepine receptors in PD. Both baclofen and progabide entered clinical trials but neither was developed [59, 60]. It could be that there is no specific target for modulating GABAergic neurons in the basal ganglia given the widespread distribution of GABAergic neurons and the related receptors throughout the brain, which in turn may lead to adverse events. One approach that may have relevance is the introduction of the gene for glutamic acid decarboxylase (GAD) into the basal ganglia using a viral vector, and this may produce alterations in motor function.

Perhaps the biggest pharmacological disappointment in attempting to develop non-dopaminergic approaches to treatment has been in the modulation of glutamatergic activity. Glutamate is the major excitatory neurotransmitter in the brain, and changes in glutamatergic neuronal activity within the striato-thalamo-cortical loops are accepted as involved in the motor symptoms of PD and also in the expression of dyskinesia, notably at the level of the subthalamic nucleus [61-64]. Glutamate receptors have been well characterised with major classes and subtypes identified and their distribution in brain maps. A range of selective glutamate antagonists have been developed, and many of these have been shown to have effects on motor function in experimental models of PD. Modulation of ionotropic (NMDA, AMPA, and kainate) and metabotropic (mGluR4, mGluR5, *etc*.) glutamate receptors and modulation of glutamate release (sodium channel blockers) have been attempted. However, it has been proven difficult to identify a receptor subtype solely localised in the basal ganglia, and given the wide distribution of these receptors in the brain, it is perhaps not surprising that a narrow therapeutic window exists in the clinical trial and adverse events are common. To date, the non-selective NMDA receptor antagonist, amantadine, remains the only approved glutamatergic drug for PD, although safinamide also has some glutamatergic effects.

## 5 ILLUSTRATION OF SOME SUCCESS

After presenting a picture of doom and gloom, it seems appropriate to look at some areas where at least limited success has been achieved. The serotoninergic system is one illustration of where a major effort to identify targets for both motor and non-motor symptoms of PD has revealed significant and perhaps clinically important drug effects. Reasons for focussing on serotonin include the following: 1) loss of serotoninergic neurons is a core component of the primary pathology of PD affecting the raphe nuclei and serotonin inputs to a range of brain areas [27]; 2) early experimental studies in which serotonin neurons have been destroyed show clear evidence of involvement in the genesis of normal motor function [65, 66], making this an attractive target; 3) more recent studies have implicated the serotoninergic system in the expression of levodopa-induced dyskinesia [67-70]; 4) serotonin has long been implicated in depression and current evidence suggests that antidepressant drugs that possess serotonin reuptake inhibitory properties may be effective in treating depression in PD [14]. But then, there is the attraction to the plethora of serotonin receptors that are potentially available as drug targets in PD. Multiple serotonin receptors have been identified along with subtypes of each of these receptors, but this may, in fact, be a complication in terms of pharmacological manipulation as these add to the complexity of the mechanisms controlling serotoninergic transmission along with their pre- and post-synaptic localisation. This can be seen in the difference in outcomes of both preclinical and clinical investigations of subtype-specific drugs for PD. However, there are at least two examples of some success.

The first relates to the observation that pharmacological manipulation of 5HT-1A serotonin receptors can reduce the intensity of levodopa-induced dyskinesia in experimental models of PD [71]. Indeed, in early clinical studies, several 5-HT-1A agonist drugs have been shown to exert anti-dyskinetic actions. However, in larger-scale phase 3 studies, these effects have not been replicated, and a worsening of motor symptoms has been observed along with dose-limiting adverse events [72]. This was unexpected based on the experimental findings, and it may relate to off-target actions of the compounds tested. For this reason, further clinical studies are currently underway to look at compounds of this class that are more specific in their interaction with 5-HT1A receptors. The second relates to drug action at 5-HT2A receptors that have been implicated in visual hallucinations in PD. Pimavanserin is a 5-HT2A inverse agonist/antagonist that is devoid of dopaminergic, histaminergic, adrenergic, and muscarinic activity. It has now been shown to improve hallucinations and delusions without worsening motor function in pivotal clinical trials and open-label extension studies in PD [73-76].

A final example relates to the development of adenosine A2 receptor antagonists, which is a story of both success and failure. Adenosine is not a classical neurotransmitter in that there are no adenosinergic neurons. Rather, adenosine is a ubiquitous metabolic intermediate formed from adenosine triphosphate, adenosine diphosphate, and adenosine monophosphate that functions as a neuromodulator released both by neurons and glial cells [77]. The interest in adenosine A2a receptors relates to their highly selective localization to the basal ganglia in man where they are present on both the cell bodies and the terminals of the indirect strio-pallidal GABAergic output pathway. As such, they are ideally localised to modulate the activity of this key pathway in both Parkinson's disease and in levodopa-induced dyskinesia. Indeed, in both rodent and primate models of PD, adenosine A2a antagonists are able to improve motor function both alone and in combination with levodopa without enhancing the intensity of established dyskinesia [29, 78-80]. The most investigated drug has been istradefylline, which has an excellent scientific base supporting its use in PD [78]. But the development of these compounds has proved to be problematic. Over the last 20 years, several A2a antagonists have been explored in clinical trials showing improvement in ‘off’ time and improvement in ‘on’ time without troublesome dyskinesias in patients with motor fluctuations, at least in phase II investigations. However, few have progressed to phase III studies because of toxicity or bioavailability issues, and the promise of early studies has not been replicated. Istradefylline was examined in 8 phase III clinical trials but in only 3 were there significant changes in ‘off’ time accompanied by an increase in ‘on’ time without troublesome dyskinesia. Based on the positive studies, istradefylline was approved for use in Japan and then in the USA, but the drug was rejected by the EU authorities based on the limited evidence of efficacy over the entire clinical program [78]. Other examples of A2a antagonists include preladenant and tozadenant. Preladenant exhibited antidepressant-like profiles and reduced L-DOPA-induced behavioural sensitisation in rodent models of PD [81]. It seems that oral preladenant is well-tolerated and associated with fewer motor complications [82]. However, when this drug was tested in PwP, neither preladenant monotherapy nor in combination with levodopa appeared useful when compared to PwP taking rasagiline [82-84]. Despite promising Phase 2 study data, tozadenant was shown to have haematological toxicity, which necessitated ceasing an ongoing phase 3 investigation of this drug [85].

This raises the important question of why the translation of what appears to be a clear target for the treatment of PD has not translated into a clinically relevant drug class despite extensive laboratory evidence that efficacy would be achieved. In the immediate case of adenosine A2a antagonists, this may relate to the design of the clinical trials which were undertaken in late-stage patients with PD on maximised dopaminergic medication despite preclinical studies showing that this drug class is most effective when administered in combination with low or threshold doses of levodopa [78]. But this is not an isolated example of the lack of translation that is occurring when trying to develop non-dopaminergic approaches to the treatment of PD. Recent papers have highlighted the failure of drugs acting through glutamatergic, serotoninergic, nicotinic, and noradrenergic mechanisms to fulfill the promise from preclinical studies of alleviating levodopa-induced dyskinesia [86, 87]. A general discussion of the reasons why drug development in the non-dopaminergic arena is problematic is given at a later point in this review.

## 6 MOVING AWAY FROM SINGLE-ACTION DRUGS TOWARDS A MULTIMODAL APPROACH

So far, highly targeted single-action drug approaches to the treatment of motor symptoms and NMS through non-dopaminergic mechanisms have not been spectacularly successful. An alternative approach is to accept that the complexity of the pathology and biochemistry of PD contributes to the array of motor and NMS that characterise their expression in the illness and opens another potential avenue for future drug development involving a change in multiple neurotransmitter pathways. This implies that molecules possessing multiple receptor and/or biochemical actions may be more effective than compounds acting on specific receptors in limited areas of the central nervous system. This goes against the general mantra of drug development within the pharmaceutical industry that seeks single action targets as means of producing an effect without off-target side-effects and might be seen as a return to the era of ‘dirty drugs’.

This is perhaps where terminology becomes important. A ‘dirty drug’ is usually defined as a molecule that has multiple pharmacological effects, many of which are off-target actions that lead to the onset of adverse events [88]. In contrast, a multimodal drug is one that also possesses a number of different pharmacological actions but which are directed towards deficits associated with the disease state [89]. In other words, a molecule with a rich pharmacology could overcome multiple biochemical changes that occur in PD without the necessity for a different drug aimed at each potential target that would lead to polypharmacy. This is not easy as introducing the right balance of pharmacological activity into a single molecule such that the desired degree of receptor activation, flexibility in dosage, and individualization of use could be achieved is a complex process. However, we already have some examples of multimodal drugs in use in PD with evidence for additional benefit over single-action drugs.

Even within the dopaminergic arena, the potential benefit of multiple pharmacological activities can be seen. For example, L-dopa is not only converted to dopamine but is further metabolised to noradrenaline and can interfere with serotoninergic transmission [90]. Drugs, such as apomorphine and rotigotine, differ from focused dopamine agonists, such as ropinirole and pramipexole, in that they have a broader spectrum of interaction with all types of dopamine receptors (D1-D5), but they also have a wide range of actions at noradrenergic and serotoninergic sites [91]. What is interesting is that these are some of the better drugs that we have for the symptomatic treatment of PD.

There may be an even advantage in combining dopaminergic activity with a non-dopaminergic mechanism. An example of this approach is safinamide, which was introduced as a reversible selective MAO-B inhibitor with a long duration of effect that was indicated for the reduction in ‘off’ time of later stage PD [29, 92-94]. However, safinamide has other activities, which include state-dependent sodium channel blockade that can lead to the reduction in presynaptic glutamate release [95]. This has been shown in *in vitro* studies, in whole animal investigations, and to some degree, in man [94-97]. This may be a more important component of the clinical use of this drug than its MAO-B inhibitory activity. In MPTP-treated primates, safinamide enhances the motor effects of L-dopa, while suppressing the expression of dyskinesia [98]. The latter effect is probably a reflection of a decrease in glutamatergic transmission as a result of safinamide’s sodium channel-blocking properties. The ability of safinamide to decrease the intensity of dyskinesia has also been demonstrated in PD in those individuals with marked to severe involuntary movements, and at higher doses where sodium channel occupation will occur [92-94]. In addition, other studies and post-hoc analysis of pivotal clinical trials have shown that safinamide may also possess activity against some NMS, most notably those associated with pain, cognitive change, and mood [99]. The difference in the spectrum of activity from other MAO-B inhibitors, such as selegiline and rasagiline, presumably reflects the rich pharmacology of safinamide.

Zonisamide is a good example of a repurposed drug, although currently not commonly used in PD; it is not dopaminergic in nature but has a range of activities relevant to the treatment of the illness. Zonisamide is a widely used anti-epileptic agent, but in Japan, it has been developed separately for the treatment of off periods in later-stage PD and the reduction of established dyskinesia [29, 100-102]. The multiple pharmacological actions of zonisamide include the inhibition of voltage-gated sodium channels, T- type calcium channels, and MAO-B. As a result, it can alter GABA, glutamate, and dopamine transmission. Further exploration of its actions in PD by wider trials is warranted as these may reveal a role for other anti-epileptic agents possessing similar rich pharmacology.

However, the archetypal ‘dirty drug’ for the treatment of PD remains amantadine. Amantadine has a 40-year proven track record as a drug able to suppress the intensity of established dyskinesia in PD, but also as a molecule that can be used for early symptomatic improvement in motor function. Amantadine is usually defined as being a weak NMDA antagonist, but the reality is that it has a complex pharmacological profile and that its mechanism of action remains unclear [29, 103-105]. In all probability, the anti-dyskinetic effects of amantadine are due to the blockade of NMDA glutamate receptors, while its ability to improve motor function relates to the actions of amantadine on the dopaminergic system, including dopamine release. However, it is quite clear that amantadine also has actions on noradrenergic, serotoninergic, and cholinergic neuronal systems [103]. Attempts to ‘clean up’ amantadine and to produce a more selective NMDA receptor antagonist have not resulted in drugs that possess the same efficacy. For example, memantine has not been found to be as effective in the treatment of PD as amantadine, but it has, of course, found a role in the treatment of some aspects of dementia [28, 106-108].

The above are just examples of how utilising molecules that have multiple pharmacological effects can be of benefit in the treatment of PD, but these are almost chance occurrences, and as far as we are aware, there has not been a detailed investigation of how molecules of this type might be useful additions to PD therapy. It also raises the question of how many molecules have been discarded as a result of having a ‘dirty drug’ profile that may, in fact, have turned out to be useful pharmacological tools in the treatment of PD. In part, this problem might be related to how “therapeutic success” is defined. It may be wondered that if “dirty drugs”, such as amantadine and safinamide, are useful according to our above paradigms, why such longstanding drugs in PD have not received more recognition and are perceived by many as only marginally useful in, for *e.g*., the treatment of dyskinesia. Part of the problem here might lie in the choice of outcome measures and the prime example would be the continued use in many trials of the Movement Disorder Society’s Unified Parkinson’s Disease Rating Scale (MDS-UPDRS) as a “gold standard”. Despite its widespread use, it has known floor effects, problems related to intra- and inter-rater reliability, and is susceptible to symptomatic treatment-related variability [109, 110], in addition to possible lack of experience of raters, time constraints during assessments, or the inability to appreciate the presence of certain symptoms [6, 111]. Moreover, many symptoms related to PD are not, or only to a limited extent, captured by this scale, including many non-motor symptoms [112]. A good example of how a drug could prove more successful after changing the primary endpoints is safinamide where evidence is accumulating for its superior analgesic effect compared to other pain therapies in PwP [113, 114], as discussed earlier.

## 7 ADVANCING DRUG THERAPY THROUGH SUBTYPES OF PARKINSON’S DISEASE

Attempting to develop non-dopaminergic approaches to the treatment of PD, particularly for the alleviation of non-motor symptoms, is proving a challenge when based on a general approach to pharmacological targets that seem relevant to PD based on the known pathology and biochemistry of the disease. Nonetheless, as outlined before, PD is a heterogeneous condition, both from a clinical as well as a neurochemical perspective, and the relative importance of different neuronal pathways and transmitter systems is likely to differ between patients and disease stages [25, 36, 115-118]. Therefore, selective therapies may still prove useful once optimal drug targets have been established.

A viable alternative strategy might be to use clinical phenotyping combined with data-driven approaches to delineate non-motor symptom dominant subgroups, also referred to as non-motor subtypes or endophenotypes of PD. These subtypes subsequently could form the basis of subtype-specific therapies and aid in the delivery of personalised medicine [51] when linked to clear biochemical and pathological patterns of disease development. Currently, several non-motor dominant subtypes, along with previously described motor subtyping [35], exist. Rodriguez-Sanchez *et al.* recently showed a multi-partition clustering method to be able to identify eight distinct subtypes, clustering around impulse control problems, overall non-motor symptoms, presence of dyskinesias and psychosis, fatigue, axial symptoms and motor fluctuations, autonomic dysfunction, depression, and excessive sweating [116]. Mu *et al.* showed that, based on motor and non-motor symptoms, cluster analyses identified four clusters: 1) mild motor and non-motor symptoms, 2) severe non-motor, but mild motor symptoms, 3) severe motor, but mild non-motor symptoms, and 4) severe motor and non-motor symptoms [34]. Within the non-motor symptoms, clustering tended to occur around urinary, neuropsychiatric, memory/cognition, and gastrointestinal symptoms [34]; in addition, there are clinically defined subtypes that are presumed to be linked to noradrenergic, cholinergic, and serotonergic changes, and hold relevance for specification of subtype-specific treatments [36, 51, 119]. An overview of some of the proposed motor and non-motor subtypes is provided in Table **2**.

The above aspect makes it clear that PD cannot be considered a homogenous disease and, in fact, is best classified as a syndrome [120]. As such, PD subtypes or endophenotypes should be taken into account when determining the optimal treatment for symptoms, effectively personalising treatment options [25]. A practical approach to this would be to consider a clinical endophenotype classification, where dysfunction in dopaminergic pathways is a major contributor to PD symptoms, but where other major neurotransmitters, such as acetylcholine, noradrenaline, and serotonin, are also involved and considered subtypes of PD dependent on dominant symptoms [25, 121]. This proposal is based on Lewy body deposition in the brain, with stage one associated with olfactory bulb involvement, as well as an anterior olfactory nucleus and the lower medulla, stage 2 involving the lower brainstem containing non-dopaminergic nuclei, such as the median raphe (serotonin) and locus coeruleus (noradrenaline), and stages 3 to 6 reflecting more widespread pathology [25, 121]. This is corroborated by studies showing greater loss of cholinergic pedunculopontine nucleus neurons and substance P-containing neurons in the dorsal motor nucleus of the vagus with relative sparing of tyrosine hydroxylase-immunoreactive neurons in the dopaminergic system [122-124]. As the dorsal nucleus of the vagus is considered crucial for autonomic signalling, individuals with PD with involvement of these centres may present primarily with autonomic symptoms [121]. Conversely, individuals with PD whose cholinergic system has been affected as part of the disease process [125-127], may have a higher risk of progression to dementia [127]. Finally, patients with a serotonergic subtype, are likely to suffer from severe fatigue and may develop levodopa-induced dyskinesia [128].

Such subtype-driven approaches would not only enable better treatment of ‘obvious’ symptoms, such as cognition in the cholinergic subtype, but would also allow for examination of more specific treatment of other symptoms associated with the specific neurotransmitter deficits in these subtypes. An example includes cholinergic neurotransmission, where Allen *et al.* have recently reviewed non-cognition-related evidence for the use of acetylcholinesterase inhibitors, and showed that such medication may reduce the rate of falls by up to 50% [50]. Moreover, Svenningsson *et al.* recently demonstrated IRL752, a novel small-molecule compound that acts to enhance norepinephrine, dopamine, and acetylcholine neurotransmission in a region-specific fashion, as safe and well-tolerated with preliminary evidence for efficacy in motivation/initiative and axial motor symptoms [93, 129]. Looking at new outcomes for acetylcholinesterase inhibitors and moving away from the paradigm set out by research on Alzheimer’s disease and the role of acetylcholine will enable us to look beyond the traditionally assigned role of this neurotransmitter in cognition. Of interest here is the observation that cholinergic nuclei in the brain may be unevenly affected in PD and that cholinergic neocortical asymmetry parallels ipsilateral dopaminergic degeneration [130]. However, before the use of cholinesterase inhibitors and before similar drugs can be further optimised for non-cognitive targets in PD, a better understanding of the relation between acetylcholine and other neurotransmitters, including dopamine, is needed.

Other subtype-driven approaches might include serotonin-norepinephrine reuptake inhibitors and tricyclic antidepressants that can significantly improve depressive symptoms [131]; these would be of particular use in the serotonergic subtype of PD [25]. Interestingly, it has been postulated that targeting serotonergic neurotransmission might also improve motor function, especially levodopa-induced dyskinesia. A PET imaging study involving PwP with motor fluctuations showed that with the use of buspirone, a partial 5-HT1A serotonin receptor agonist, putaminal dopamine peaked less following the intake of levodopa [70]. In addition, several small clinical studies have shown improvements in dyskinesia with partial 5-HT1A receptor agonists, such as buspirone, sarizotan, and tandospirone [132-134]. Not only for motor symptoms would the serotonergic system be of importance, but certainly also for non-motor symptoms. Although mostly linked to symptoms such as pain, autonomic, and neuropsychiatric symptoms, such as apathy and depression, the exact role of serotonergic neurotransmission has not been fully unraveled [135]. The promise of this neurotransmitter pathway might lie in targets traditionally associated with hyperdopaminergic states, such as hallucinations. An example of this is pimavanserin, a selective 5-HT2A receptor inverse agonist/antagonist, used to treat hallucinations and delusions occurring in PwP [135], as also discussed before.

Other targets that might prove beneficial in relation to the discovery of other subtypes of PD include histamine and γ-aminobutyric acid (GABA). The involvement of the histaminergic system in PD has been shown in several studies [136-138], and preclinical studies have shown that H3R antagonist/inverse agonists, such as thioperamide, may alleviate apomorphine-induced behavioural responses in rats and improve memory impairments in rodent models [139, 140]. Adenosine, a ubiquitous metabolic intermediate formed from adenosine triphosphate, adenosine diphosphate, and adenosine monophosphate, functions as a neuromodulator, released both by neurons and glial cells [77]. The involvement of GABA neurotransmission has been shown in PD [30]. As dopamine-releasing neurons in the substantia nigra pars compacta inhibit striatal neurons through postsynaptic activation of GABA receptors [57], targeting the GABAergic system might prove beneficial in improving dopaminergic neurotransmission. In preclinical models, geraniol, binding to GABA receptors, attenuated α-synuclein expression in MPTP-treated mice [141], as well as improved the motor phenotype of this PD model [142].

## CONCLUSION

**Fig. (1):**
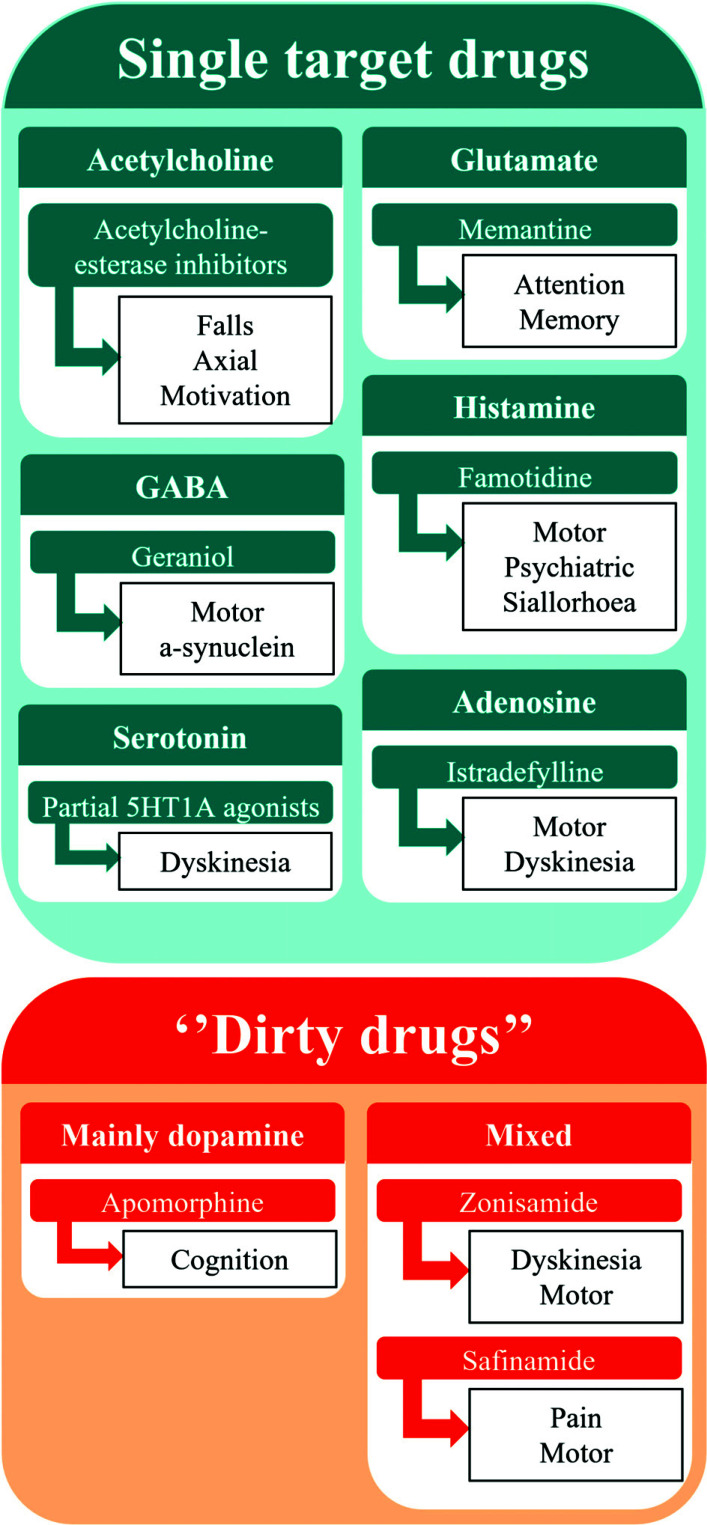
Expected pharmacological non-dopaminergic targets for further development in Parkinson’s disease.

Despite extensive laboratory investigations and a range of clinical trials that have tried to identify novel treatments for PD, the currently available arsenal of pharmacological interventions is relatively limited and heavily biased towards dopaminergic replacement or modifications of its metabolism. In part, the failure to produce new treatments might be due to the difficulties in translating preclinical findings into clinical research. As such, motor complications and non-motor symptoms in PwP remain a challenge in the successful treatment of PD, but substantial preclinical and clinical evidence is becoming available that the use of optimised dopamine-based regimes, as well as compounds targeting non-dopaminergic neurotransmitter and neuropeptide systems, can be useful. Several of the targets that have been discussed here are relevant and can be developed further to optimise symptom-control in PwP. Nonetheless, as we have highlighted, several barriers need to be overcome in the translation of preclinical findings to clinical research. These barriers include the careful selection of preclinical models, clinical study populations, targets, and outcomes, reflecting the heterogeneous nature of PD [25, 34]. Drugs acting broadly on several pathways and targets may be more efficacious than selective compounds, yet given the heterogeneity of PD, complimentary targets in addition to the ‘common base’ may be necessary to offer personalised treatments and improve quality of life on an individual basis [143]. We feel that future developments should move away from dopamine-based compounds and target non-dopaminergic systems instead. Promising candidate compounds in this respect are ‘dirty drugs’, such as amantadine, safinamide, and zonisamide, but also acetylcholinesterase inhibitors and serotonin-based strategies (Fig. **1**).

## Figures and Tables

**Table 1:** Examples of symptoms of Parkinson’s disease and level of control.

**What we can Control Reasonably**	**What we Cannot Control Reasonably or Only Within Certain Limits**
Bradykinesia and rigidity	Tremor
Peak dose and aspects of diphasic dyskinesia	On-related freezing
Motor fluctuations	Falls
Dopamine-responsive non-motor symptoms *Depressive symptoms* *Anxiety* *Drowsiness* *Impulse control disorders* *Constipation* *Urinary urgency* *Fluctuation-related pain*	Camptocormia/axial dystonia
	Unpredictable dyskinesia
	Dopamine unresponsive non-motor symptoms *Cognitive dysfunction* *Visual disturbances* *Orthostatic hypotension* *Fatigue* *Apathy* *Other types of pain*

**Table 2:** Proposed motor and non-motor subtypes of Parkinson’s disease.

**Subtype Proposed**	**Number of Subtypes**	**Basis of Classification**	**References**
• Mild motor dominant • Diffuse motor dominant • Malignant motor dominant	3	Predominantly motor	[35]
• Tremor-dominant • Indeterminate • Postural instability and gait	3	Predominantly motor	[144, 145]
• Tremor-dominant • Mixed • Akinetic-rigid	3	Predominantly motor	[146-148]
• Tremor-dominant • Akinetic-rigid • Gait difficulties • Mixed	4	Predominantly motor	[149]
• Fast motor • Mild motor and non-motor • Severe motor • Slow motor	4	Mixed motor and non-motor	[150]
• Mild motor and non-motor symptoms • Severe non-motor, but mild motor symptoms • Severe motor, but mild non-motor symptoms • Severe motor and non-motor symptoms	4	Mixed motor and non-motor	[34]
• Impulse control problems • Overall non-motor symptoms • Dyskinesias and psychosis • Fatigue • Axial symptoms and motor fluctuations • Autonomic dysfunction • Depression • Excessive sweating	8	Mixed motor and non-motor	[116]
• Brainstem • Limbic • Cortical	3	Predominantly non-motor	[36]
• Cognition • Apathy • Depression/Anxiety • Sleep • Pain • Fatigue • Autonomic	7	Predominantly non-motor	[25, 51]
• Cholinergic • Serotonergic • Noradrenergic • Mixed	4	Predominantly non-motor	[25, 51]
• Mild baseline moderate progression • Moderate baseline mild progression • Severe baseline rapid progression	3	Mixed motor and non-motor	[118]

## LIST OF ABBREVIATIONS

GABA: γ-Aminobutyric Acid
GAD: Glutamic Acid Decarboxylase
NMS: Non-motor Symptoms
PD: Parkinson’s Disease
